# Supplementary material for: Identification of inhibitors that target dual-specificity phosphatase 5 provide new insights into the binding requirements for the two phosphate pockets
Source: BMC Biochem. 2015 Aug 19;16:19. doi: 10.1186/s12858-015-0048-3 (PMC4545774; doi:10.1186/s12858-015-0048-3)
Supplement: Additional file 1: — Supplementary Figures. (DOCX 401 kb) [file 12858_2015_48_MOESM1_ESM.docx]

Identification of inhibitors that target dual-specificity phosphatase 5 and provide new insights into the binding requirements for the two phosphate pockets

Terrence S. Neumann[1,2], Elise A. Span[2], Kelsey S. Kalous[2], Robert Bongard[2], Adam Gastonguay[3], Michael Lepley[3], Raman Kutty[3], Jaladhi Nayak[3], Chris Bohl[2], Rachel G. Lange[2], Majher I. Sarker[4], Marat R. Talipov[4], Rajendra Rathore[4†], Ramani Ramchandran[3†], and Daniel S. Sem[2†$]

Author Affiliations and Addresses:

[1]Department of Chemistry and Biochemistry, Texas Wesleyan University, 1201 Wesleyan Ave., Fort Worth, TX 76105 USA, [2]Center for Structure-based Drug Design and Development, Department of Pharmaceutical Sciences, and School of Pharmacy, Concordia University of Wisconsin, 12800 N. Lake Shore Drive, Mequon, WI 53097, USA, [3]Department of Pediatrics, Department of Obstetrics and Gynecology, Medical College of Wisconsin, Children's Research Institute (CRI) Developmental Vascular Biology Program, Translational and Biomedical Research Center, Milwaukee, WI 53226, USA, [4]Department of Chemistry, Wehr Chemistry Building, P.O. Box 1881, 535 N. 14th Street, Milwaukee, WI 53201, USA.

[†] These authors contributed equally to this work

[$]**Corresponding Author and Address:** Daniel S. Sem, Ph.D. Center for Structure-based Drug Design and Development, Department of Pharmaceutical Sciences, School of Pharmacy, Concordia University Wisconsin, 12800 North Lake Shore Drive, Mequon, WI 53097, USA. Email: daniel.sem@cuw.edu, Phone: 262-543-2778

Emails: Terrence S. Neumann (tneumann@txwes.edu), Elise A. Span (elise.span@gmail.com), Kelsey S. Kalous (kekalous@mcw.edu), Robert Bongard (Robert.bongard@cuw.edu), Adam Gastonguay (agastong@mcw.edu), Michael Lepley (mlepley@mcw.edu), Raman Kutty (rkutty@mcw.edu), Jaladhi Nayak (jnayak@mcw.edu), Chris Bohl, Rachel G. Lange, Majher I. Sarker (majher.sarker@ars.usda.gov), Marat R. Talipov (marat.talipov@marquette.edu), Rajendra Rathore (rajendra.rathore@marquette.edu), Ramani Ramchandran (rramchan@mcw.edu), and Daniel S. Sem (daniel.sem@cuw.edu)

Abstract

Background: Dual-specificity phosphatase-5 (DUSP5) plays a central role in vascular development and disease. We present a *p*-nitrophenol phosphate (pNPP) based enzymatic assay to screen for inhibitors of the phosphatase domain of DUSP5.

Methods: pNPP is a mimic of the phosphorylated tyrosine on the ERK2 substrate (pERK2) and binds the DUSP5 phosphatase domain with a K_m of 7.6 $\pm$ 0.4 mM. Docking followed by inhibitor verification using the pNPP assay identified a series of polysulfonated aromatic inhibitors that occupy the DUSP5 active site in the region that is likely occupied by the dual-phosphorylated ERK2 substrate tripeptide (pThr-Glu-pTyr). Secondary assays were performed with full length DUSP5 with ERK2 as substrate.

Results: The most potent inhibitor has a naphthalene trisulfonate (NTS) core. A search for similar compounds in a drug database identified suramin, a dimerized form of NTS. While suramin appears to be a potent and competitive inhibitor (25 $\pm$ 5 μM), binding to the DUSP5 phosphatase domain more tightly than the monomeric ligands of which it is comprised, it also aggregates. Further ligand-based screening, based on a pharmacophore derived from the 7 Å separation of sulfonates on inhibitors and on sulfates present in the DUSP5 crystal structure, identified a disulfonated and phenolic naphthalene inhibitor (**CSD^3_2320)** with IC_{50} of 33 μM that is similar to NTS and does not aggregate.

Conclusions: The new DUSP5 inhibitors we identify in this study typically have sulfonates 7 Å apart, likely positioning them where the two phosphates of the substrate peptide (pThr-Glu-pTyr) bind, with one inhibitor also positioning a phenolic hydroxyl where the water nucleophile may reside. Polysulfonated aromatic compounds do not commonly appear in drugs and have a tendency to aggregate. One FDA-approved polysulfonated drug, suramin, inhibits DUSP5 and also aggregates. Docking and modeling studies presented herein identify polysulfonated aromatic inhibitors that do not aggregate, and provide insights to guide future design of mimics of the dual-phosphate loops of the ERK substrates for DUSPs.

Keywords: DUSP5; phosphatase; drug discovery; docking; suramin; vascular anomalies

Background

Mitogen-activated protein kinases (MAPKs), such as extracellular regulated kinase (ERK) [1], are activated by phosphorylation of tyrosine and serine/threonine residues in their activation loops. MAPKs can then be deactivated by phosphatases that remove these phosphate groups from their activation loop. One such class of phosphatases, dual-specificity phosphatases (DUSPs), is unique in that it can dephosphorylate both serine/threonine and tyrosine residues. The Ramchandran lab has shown that DUSP5 is necessary for early vascular patterning in vertebrates, and is mutated in patients with vascular anomalies [2]. DUSP5 plays a regulatory role in vascular development based on its ability to specifically interact with and dephosphorylate phosphorylated ERK (pERK) [3-7]. Indeed, we identified a clinically relevant serine to proline mutation (S147P) that is associated with vascular defects [2]. This mutation has been shown previously by our group to interfere with the dephosphorylating activity of DUSP5 protein [8], and makes the protein hypoactive. However, the direct causal role of S147P in vascular anomaly progression is yet to be established. Nevertheless, DUSP5 is a critical drug target for vascular-related diseases, and more broadly, MAPKs and their DUSP partners are involved in cell signaling that is directly involved in a wide range of diseases, including cancer, diabetes, and autoimmune disorders [6,9-11]. Recently, DUSP5 has gained increased attention in the scientific literature [12,13,14] especially as it relates to loss or gain of expression of DUSP5 in murine models, and its associated phenotypic changes in both the immune and cancer biology systems. DUSP5 knockout (KO) mice are alive, and display no overt phenotype, indicating that it is dispensable for embryonic development. However, Holmes et al did report that these mice showed increased function and survival of eosinophils, a key player in the immune system's ability to clear parasitic infections [12]. Further, Rushworth *et al* reported increased sensitivity to a skin cancer model in their murine model [14]. In terms of the vasculature, the DUSP5 KO rat displays enhanced myogenic response and autoregulation of cerebral blood flow [15]. Taken together, these studies demonstrate that inhibition of DUSP5 will result in biologically relevant changes *in vivo*.

From a conformation perspective, DUSP5 is comprised of two domains, an N-terminal ERK binding domain (EBD) and a C-terminal phosphatase domain (PD) [5,16]. While there is no structure available for intact DUSP5, there is a crystal structure of the PD [162]. The DUSP5 PD structure has two anionic sulfate groups bound in the active site near the catalytic Cys263 (mutated to serine in the structure), and separated by 7.2 Å. These sulfates had been proposed to occupy the same binding pockets that are occupied by the phosphate groups on the substrate [16]. For the ERK2 substrate, the pThr-Glu-pTyr tripeptide region of the ERK2 activation loop presumably occupies this region in the DUSP5 PD [17, 18].

A molecular model based on the crystal structures of the human DUSP5 PD and ERK2, and a homology model of the DUSP5 EBD, illustrates the bivalent nature of the interaction between the two domains of DUSP5 and the ERK2 substrate (Figure 1) [8]. The current study has identified small molecule inhibitors that occupy the active site pocket on the DUSP5 PD, and act as inhibitors of the phosphatase enzymatic activity. We further tested some of these compounds as inhibitors of full-length DUSP5 and in *in vitro* assays, to relate inhibition results and conclusions to more biologically and clinically relevant situations.

Methods

Molecular docking

The Center for Structure-based Drug Design and Development (CSD[3]) chemical library, consisting of 11,500 drug-like chemicals, was prepared in electronic format as two-dimensional (2D) SDF files. Using Pipeline Pilot [23], the protonation state of all compounds was adjusted to reflect the most prevalent form at a pH of 7.4. CORINA [24] was used to convert these files to three-dimensional (3D) PDB coordinate files, which resulted in energy-minimized 3D structures. The files were then processed with the python script prepare_ligand4.py, which comes with the Autodock Tools Suite [25]. This script generates a pdbqt file and adds partial charges to the ligand, sets all torsions in the ligand to active (to permit rotation), and merges all non-polar hydrogen atoms.

The DUSP5 PD structure (PDB:2G6Z) [16] was prepared for docking using the Autodock Tools Suite [25]. Grid maps were used in the energy calculations performed by Autodock. Partial charges were added and all non-polar hydrogen atoms were merged, resulting in a pdbqt file. The 13 different grid maps, one for each of the different atoms present in the chemical library of compounds (ex. C, H, F, Cl, etc.), were generated using Autogrid4 [25]. A grid box, the site used to dock the ligands, was positioned to cover the entire protein in a blind docking experiment to ensure unbiased identification of binding location and orientation.

The docking parameter file (dpf), which contains the parameters that Autodock4 uses to dock ligands into the protein, was prepared using the python script prepare_dpf4.py, and default docking parameters were used, except that 50 separate docking calculations were performed with each calculation consisting of 1,750,000 energy evaluations, and a root mean square deviation (rmsd) tolerance set to 2.0 angstroms (to define entry of structure into a given cluster). The dpf files were then automatically docked using the MUGrid Cluster (Marquette University) with HTCondor [26,27] and AutoDock4 [25,28] using the Lamarckian genetic algorithm local search method to perform the optimization of docking poses. The docking poses were then clustered on the basis of the rmsd between the coordinates of the atoms in a given ligand, and were ranked on the basis of calculated free energy of binding. The docking log files were then analyzed using the python script summarize_results4.py contained in the shell script sumresults_4.py [25], which rank orders all the dockings by binding energy. The results were then analyzed to find the best-clustered compounds with lowest free energy of binding as determined by Autodock4.2. Additional docking of all experimentally tested chemicals was performed as described above, but with 100 dockings trials.

Ligand-based searching

As previously described, the CSD[3] chemical library was electronically prepared and protonation state adjusted using Pipeline Pilot [23]. Using OpenEye Scientific Software's Omega2 [29,30], three dimensional coordinates were calculated and stored in OpenEye Scientific Software's preferred file format, .oeb.gz, for subsequent molecular overlay evaluation. OpenEye Scientific Software's Rapid

Overlay of Chemical Structures (ROCS) [29] software was used to search for molecules with similar shape and electronic properties to a lead molecule. Lead molecules identified from DUSP5 docking and inhibition studies were used as chemical queries to search a database of Food and Drug Administration (FDA) approved drugs, to identify FDA approved drugs that might also be DUSP5 inhibitors.

The ZINC library [31] of 13 million commercially available chemicals was obtained as 2D SDF files and prepared similarly to the CSD[3] chemical library for use with OpenEye Scientific Software's ROCS. This further expanded the availability of chemical analogs available for experimental screening. ROCS calculations were also performed against the DrugBank [32,33] database of FDA approved drugs.

Synthesis of RR505 and RR506. Synthesis of Carbazole-1,3,6-trisulfonic acid, trisodium salt (RR505)

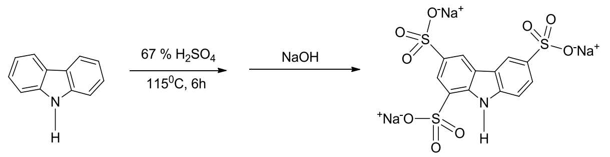

Solid carbazole (3.0 g, 17.9 mmol) was placed in a 50-mL round-bottom flask and 67% H_2SO_4 (12 mL) was added drop-wise at 22 °C and a slurry thus obtained was stirred and heated at 115 ± 5 °C for 6 h. The resulting dark solution was cooled to room temperature and poured into a saturated NaCl solution (100 mL) containing NaOH (2.4 g, 60 mmol) to afford an ash-colored precipitate, which was filtered, washed with saturated NaCl solution (50 mL) and dried at 90 °C for 10 h to get 7.5 g of the crude product.

The crude solid was dissolved in distilled/deionized water (150 mL), treated with activated charcoal Norit (1.1 g) and the resulting mixture was refluxed for 15 min. The solution was filtered hot through a pad of Celite®, and evaporated slowly to afford a white powder of **RR505** (5.5 g, 65 % yield). ^{1}H-NMR (400 MHz, D_2O): 7.60 (1H, d, J = 8.8 Hz), 7.79 (1H, d, J = 8.8 Hz), 8.08 (1H, s), 8.53 (1H, s), 8.64 (1H, s), ^{1}H-NMR (400 MHz, DMSO-d_6): 7.58-7.66 (2H, m), 7.96 (1H, s), 8.24 (1H, s), 8.26 (1H, s),

10.81 (1H, s). ^{13}C-NMR (100 MHz, D_2O): 112.3, 118.8, 121.6, 121.64, 121.7, 124.5, 124.7, 125.5, 133.7, 134.7, 136.9, 142.1.

Synthesis of Carbazole-1,3,6,8-tetrasulfonic acid, tetrasodium salt (RR506)

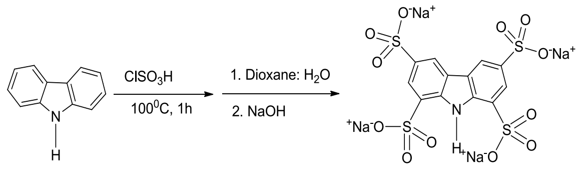

Solid carbazole (3.0 g, 17.9 mmol) was placed in a 50-mL round-bottom flask and chlorosulfonic acid (41.7 g, 358 mmol) was added in small portions with vigorous shaking at 22 ^{o}C, after which the mixture was stirred and heated at 100 ± 5 °C for 1 h. The resulting dark solution was cooled to room temperature and then poured slowly onto crushed ice (~100 g). The resulting precipitate was filtered by gravity filtration and was dried by placing between paper towels. The resulting semi-dried solid was dissolved in ethyl acetate (150 mL), treated with Norit (1.5 g), and refluxed for 15 min and filtered hot through a pad of silica gel (~1x1.8 inch). The filtrate was concentrated *in vacuo* and recrystallized from a 1:9 mixture of ethyl acetate and hexanes to afford a yellow solid, which was filtered and dried *in vacuo*.

The dried solid was dissolved in a mixture of dioxane (20 mL) and distilled/deionized water (20 mL) and heated under reflux for 12 h. The resulting solution was cooled to room temperature and was extracted with diethyl ether (2 x 50 mL) to remove nonpolar impurities. The aqueous layer was neutralized by a dropwise addition of NaOH solution (1 M) with continuous monitoring of pH using pH paper. The resulting solution was concentrated to ~10 mL and acetone was added to afford a white powder of **RR506** (3.5 g, 22 % yield, average yield from 3 runs). ^{1}H-NMR (400 MHz, D_2O): 8.12 (2H, s), 8.68 (2H, s), ^{1}H-NMR (400 MHz, DMSO-d_6): 7.97 (2H, s), 8.26 (2H, s), 10.61 (1H, s); ^{13}C-NMR (100 MHz, D_2O): 121.9, 122.1, 123.8, 125.7, 134.7, 136.7.

Alternative synthesis of RR506

Solid carbazole (1.0 g, 6 mmol) and nitrobenzene (20 mL) were placed in a 50-mL round-bottom flask and chlorosulfonic acid (14 g, 120 mmol) was added in small portions at 22 ^{o}C, after which the

mixture was stirred at 22 °C for 72 h. The resulting solution was poured into aqueous saturated NaCl solution (100 mL) containing NaOH (0.96 g, 24 mmol) which resulted in a fluffy precipitate. The precipitate thus formed was filtered and dried. The solid was dissolved in distilled/deionized water (100 mL) and refluxed with 1.5 g of Norit for 15 min and filtered hot through a pad of celite. The filtrate was concentrated to ~25 mL and **RR506** was precipitated by addition of acetone. The precipitate was filtered and dried to afford **RR506** as a white solid (2.9 g, 84 % yield). ^{1}H-NMR (400 MHz, D_2O): 8.12 (2H, s), 8.68 (2H, s), ^{1}H-NMR (400 MHz, DMSO-d_6): 7.97 (2H, s), 8.26 (2H, s), 10.60 (1H, s).

Protein production

The DUSP5 PD gene was synthesized by Blue Heron (Bothell, WA) in both an active wild type form (DUSP5 PD(WT)) and an inactive form, where the catalytic cysteine was mutated to a serine (DUSP5 PD(C263S)). The genes were inserted into Origene pEX plasmids with ampicillin resistance and an N-terminal hexa-histidine tag to facilitate protein purification. Plasmids were transformed into BL21(DE3) cells (Invitrogen) for expression.

For unlabeled DUSP5 PD(WT) preparation, an overnight culture was used to inoculate 2 L of LB (Luria-Bertani) media, containing 50 μg/mL of ampicillin. Cells were grown at 37 °C to an OD_{600} of 0.7 and then induced with 0.6 mM isopropyl β-D-1-thiogalactopyranoside (IPTG) for 4 h at 37 °C, then for 14 h at 16 °C. Cells were harvested using centrifugation and frozen prior to purification. Thawed cells were lysed in a buffer containing 50 mM Tris, 300 mM NaCl, 5 mM imidazole, and 10% glycerol at pH 7.8. Lysate was centrifuged at 15,000 rpm for 1 h. The supernatant was loaded on to Ni-Sepharose Fast Flow resin (GE Healthcare) and washed three times successively with 5 column volumes of lysis buffer containing 25 mM imidazole. Protein was eluted with lysis buffer containing 305 mM imidazole. Protein was then dialyzed in a buffer containing 50 mM potassium phosphate and 2 mM dithiothreitol (DTT) at pH 6.8.

For ^{15}N-labeled DUSP5 PD(C263S) preparation (for NMR titrations), an overnight culture was used to inoculate 2 L of LB media supplemented with 50 μg/mL of ampicillin. Cells were grown to an OD_{600} of 0.7 at 37 °C, then harvested and washed with M9 minimal media (pH 7.0) [30]. Cells were

resuspended in 500 mL M9 minimal media containing 0.5 g $^{15}NH_4Cl$, 2 g D-glucose, 5 mL Basal Medium Eagle with Earle's salts and sodium bicarbonate (Sigma Aldrich), 0.146 g L-glutamine (Sigma Aldrich) 1.0 mL 1M $MgSO_4$, and 0.5 mL 1 M $CaCl_2$ (pH 7.2) [34]. Additionally, a metal mix containing Zn, Mn, Cu, Co, B, and Mo salts was added to supply cells with necessary micronutrients [34]. Cells were allowed to acclimate for 30 min at 37 °C, then induced with 1 mM IPTG for an additional 4h at 37 °C. Cells were harvested and ^{15}N-labeled protein was purified as described before with the addition of 2 mM DTT during all purifications steps.

***p*-nitrophenol phosphate (pNPP) activity assay**

To measure enzymatic activity of the DUSP5 PD and the inhibitory capacity of selected molecules, an *in vitro* phosphatase assay was developed based on previous studies [35]. In this assay, DUSP5 PD will dephosphorylate the substrate *p*-nitrophenol phosphate (pNPP, Sigma Aldrich), yielding *p*-nitrophenolate, which absorbs at 405 nm with an extinction coefficient of 18,000 M^{-1} cm^{-1}.

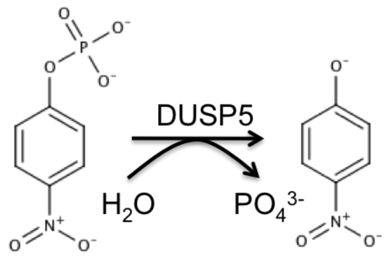

Thus, an increase in absorbance at 405 nm corresponds to the turnover of pNPP to *p*-nitrophenolate. The assay was initially optimized in 1 mL quartz cuvettes, then was subsequently optimized for and validated in a 96-well plate format. All IC_{50} values were obtained using the 96-well plate assay format (see below). The assay buffer contained 100 mM Tris, 100 mM sodium chloride, 5 mM magnesium chloride, and 1 mM DTT at pH 7.5. The pNPP substrate was prepared as a 50 mM stock by dissolving the solid substrate in assay buffer. The DUSP5 PD and pNPP were assayed initially in a cuvette (1 mL total volume) and initial velocities were fitted to the Michaelis-Menten equation:

(1) $$v = \frac{V_{\max}[S]}{K_m + [S]}$$

where v is the initial velocity, $V_{\max}$ is the maximum velocity, K_m is the Michaelis constant, and [S] is the concentration of pNPP. Data were fitted using a nonlinear least squares fit to eq. 1, with GraphPad Prism 6 software.

Validation of pNPP assay for high throughput screening (HTS)

For the 96-well plate validation assay, sodium orthovanadate (Sigma Aldrich) was utilized as a positive control for inhibition [36] at a final concentration of 10 μM, to completely block DUSP5(WT) enzymatic activity. All plate assays were performed in standard 96-well clear bottom plates (Thermo Scientific Nunc) with a total assay volume of 200 μL, using a SpectraMax M5 Microplate Reader (Molecular Devices). The plate validation assay was performed with replicate columns of positive control wells, negative control wells and blank wells. Blank columns contained only buffer and pNPP. Negative control (uninhibited) contained buffer, pNPP, and DUSP5 PD(WT); and, positive control contained the same components, but also contained 10 μM sodium orthovanadate. The plate was then shaken and allowed to equilibrate in the spectrophotometer at 25 °C for 30 min. After incubation, 4 μL of a 50 μM enzyme stock was dispensed into appropriate wells utilizing a single-channel pipette. This produced a final enzyme concentration of 1 μM. Before a read was taken, the plate was shaken for five seconds. The initial rate for the DUSP5 PD(WT) reaction was linear for approximately 90 min; and, the plate was kept in the spectrophotometer at 25 °C for an additional 80 min after the kinetic read. The endpoint reading was subsequently taken at 90 min after initiation of reaction.

Slopes from the kinetic read, as well as single-point absorbance values at the 90-minute endpoint read, were then averaged. For blank wells and positive control wells, both slope values (continuous assay) and single point absorbance values (fixed time assay) were approximately zero, as expected (Table 2).

Standard deviations were calculated and a Z' value [37] subsequently determined using the following equation:

$$(2)\quad Z' = 1 - \frac{3(\sigma_p + \sigma_n)}{|\mu_p - \mu_n|}$$

where σ_p is the standard deviation for the positive control, σ_n is the standard deviation for the negative control, μ_p is the mean for the positive control, and μ_n is the mean for the negative control. The Z' value is a coefficient denoting the quality of a high throughput screening assay, reflecting both the variation in data and dynamic range for the assay. A good assay exhibits a high signal to background ratio. A Z'-factor of 1.0 reflects an ideal assay; and, for an assay to be considered reliable, must exceed 0.5 [37].

IC_{50} measurements

IC_{50} values were obtained using the assay described above, in 96 well plates. The maximum inhibitor concentration screened in any plate was 300 mM and the minimum screened concentration was 1 μM. The IC_{50} plate was designed so that the first column of wells served as blanks, with wells containing only buffer and substrate. The second column of wells functioned as the plate negative control, with each well containing buffer, substrate and enzyme. The remaining wells in the plate contained buffer, substrate, enzyme, and varying amounts of inhibitor, with inhibitor concentration increasing from left to right across the plate. Data points were collected at a minimum in triplicate, and inhibitor concentrations were chosen to provide data equally spread on a logarithmic scale. The composition of buffer and the concentrations of substrate and enzyme utilized were identical to those in the plate validation assay. After initiation and shaking, a ten-minute kinetic read was taken.

For each plate assayed, the slope values for all negative control wells were averaged and the measured value considered representative of full enzymatic activity. Fractional activity was then calculated by dividing the slope of each inhibitor well by this value, determining the relative amount of

enzyme activity observed at each concentration of inhibitor. Values were then plotted as percent activity versus the log of the concentration of inhibitor, and fitted to the following equation:

$$(3)\ y = Bottom + \frac{(Top - Bottom)}{1 + 10^{x - \log IC_{50}}}$$

where *Top* and *Bottom* are plateaus for the values of initial velocity when uninhibited and fully inhibited, respectively.

Nephelometry

Nephelometry is a technique for measuring the relative aggregation of particles in solution, based on the light-scattering properties of molecular aggregates [38]. We performed nephelometry to explore the ability of the chemicals studied herein to form aggregates, which can lead to artifactual inhibition effects. Compounds were tested for aggregation in 96-well plates using a buffer containing 100 mM Tris base, 100 mM sodium chloride, and 5 mM magnesium chloride at pH 7.5. Each compound analyzed in these experiments contained concentrations of compound ranging from 10-100 µM, recorded in quadruplet. Each plate was analyzed at two separate gain values of 52 and 72. Data were collected using a BMG NEPHELOstar Plus, equipped with a 635 nm laser.

NMR binding assay

NMR samples of DUSP5 PD(C263S) were prepared for 2D ^{1}H-^{15}N HSQC (heteronuclear single quantum coherence) spectral titration studies. The ^{15}N-labeled DUSP5 PD(C263S) protein was concentrated using an Amicon Ultra-4 centrifugal device (Millipore) to 600 µM. NMR samples were prepared with the following conditions for **RR505**: 250 µM **RR505**, 250 µM DUSP5 PD(C263S), 10% D_2O, 50 mM potassium phosphate, 100 mM KCl, and 2 mM DTT at pH 6.8 and for **CSD^3-2320**: 0 or 500 µM **CSD^3-2320**, 500 µM DUSP5 PD(C263S), 10% D_2O, 50 mM potassium phosphate, 100 mM KCl, and 2 mM DTT at pH 6.8. NMR experiments were performed on a 500 MHz Varian NMR System using a triple resonance probe with z-axis gradients at 25 °C.

ERK dephosphorylation assay

For this assay, 10 ng of GST-tagged recombinant phosphorylated ERK2 (R&D Systems, 1230-KS) was incubated with and without the indicated DUSP5 proteins (0.5 nM final concentration) for 15 min at room temperature, with or without the indicated drugs. The reactions were halted with 2x Laemmli sample buffer and subjected to SDS-PAGE. The proteins were transferred to polyvinylidene difluoride (PVDF) and immunoblotted using antibodies to pERK (Cell Signaling Tech., #9106) and total ERK, which includes both phosphorylated and unphosphorylated ERK1 and ERK2 (Cell Signaling Tech., #9102). Bound antibodies were visualized using horseradish peroxidase-linked anti-mouse IgG (Cell Signaling Tech, #7076S) and anti-rabbit IgG (Cell Signaling Tech, #7074S), respectively, and ECL reagents (Pierce, #34708) according to the manufacturer's protocol. For calculating IC_{50} values, gel bands were imaged by chemiluminescence with either film or digital image capture by a FluorChem HD2 imager (Alpha Innotech). Density of each band was quantified with ImageJ software by using the gel analysis tool. Relative values of phosphorylated ERK present for each drug concentration treatment compared to pERK only controls were calculated. These relative values were then used to obtain IC_{50} values with GraphPad Prism 6 software. Each experiment was repeated at least three independent times, and IC_{50} values provided as a range.

Results

Docking and ligand-based *in silico* searches yield candidate small molecules that target the DUSP5 PD domain

In this study, we were interested in identifying inhibitors that could selectively target dual-specificity phosphatase 5 (DUSP5), which we have shown previously to be mutated in patients with vascular anomalies. As shown in Figure 1A, DUSP5 contains two domains namely an ERK-binding domain (EBD) and a phosphatase domain (PD) that are fused together by an unstructured linker region. The X-ray structure of PD of human DUSP5 was previously reported (PDB:2G6Z) [16], while the

structure of EBD was constructed using homology modeling based on the solution structure (21% identity and 35% homology) of human MKP-3 protein (PDB:1HZM) as a template [19]. The 30 amino acid linker region connecting the two domains, which is of unknown structure, was prepared manually. A model of the human DUSP5-ERK2 complex (Figure 1B) illustrates how DUSP5 (blue) wraps around ERK2 (yellow), its natural substrate, with the EB and PD DUSP5 domains located on opposite sides of ERK2. The model was prepared as described in our previous paper [8], and the relative orientation of ERK2 and DUSP5 is based on molecular dynamics simulations described previously [8].

In order to identify inhibitors for DUSP5, we performed *in-silico* docking of 11,500 chemicals from the CSD[3] in-house collection into the PD domain of DUSP5. The docking procedure produced a rank-ordered list of compounds that were tested using the pNPP assay (discussed below). One promising compound, **SM1842** – a trisulfonated carbazole, displayed attributes associated with lead-like chemicals (e.g. molecular weight; LogP) [39]. The ^{1}H NMR spectrum of the commercially sourced **SM1842** sample did not match the expected signal pattern for trisulfonated carbazole (Figure S1), and therefore this compound was resynthesized and its spectrum was compared with the spectrum of commercial **SM1842**. The resynthesized compound, **RR505** (Table 1), displayed the expected ^{1}H NMR spectrum for the trisulfonated carbazole. An additional synthesis of a tetrasulfonated carbazole **RR506** (i.e. an extra sulfonate, relative to **SM1842**, Table 1) and comparison of its ^{1}H NMR spectrum with that of the commercial **SM1842** demonstrated that the commercial sample was (largely) a mixture of **RR505** and **RR506**, i.e. the tri- and tetrasulfonated carbazoles (see Table 1). Further experimental analysis made use of only pure samples, i.e. **RR505** and **RR506**.

The docking pose from the lowest energy cluster for **RR505 (SM1842)** had a calculated binding energy of -9.69 kcal/mol and a cluster size of 10 (Figure 2A). Using the chemical structure of **RR505** as a search template, additional chemical libraries were computationally screened to identify related structures that could also be tested. One such compound, naphthalene trisulfonate (Table 1, NTS), was identified from the in-house collection of chemicals as well as from the ZINC collection [31] of commercially available chemicals. This compound was identified using ROCS [30], which matches

chemical queries to compounds in chemical libraries based on molecular shape and electrostatic properties. Figure 2B shows the overlay of **RR505** and NTS, using ROCS. NTS docked similarly to **RR505** in the DUSP5 PD active site pocket (Figure S2A). Interestingly, the lowest energy poses for NTS (-8.48 kcal/mol, cluster population of 7, Figure S2B) and **RR505** show a flipped binding mode relative to each other (Figures 2C and D). We hypothesize based on the ROCS alignment (Figure 2B) that the docking algorithm would position the ligands similarly. While this is not the case for the lowest energy cluster, it is the case for the second lowest energy cluster. Similarly, **RR506** was flipped relative to the lowest energy cluster pose of **RR505** (Figure S2C). And again, the second lowest energy cluster pose for RR506 (Figure S2D) matched that of the lowest energy pose of **RR505**.

Expression, purification and assay of the DUSP5 PD domain

To assess the activity of the identified compounds from *in-silico* docking, we expressed and purified the phosphatase domain of DUSP5 protein referred to as DUSP5 PD(WT) (Figure S3), and tested it in an enzymatic assay with *p*-nitrophenyl phosphate (pNPP) as a substrate. Protein was expressed in *E. coli* and purified as described above. SDS-PAGE gel analysis of DUSP5 PD(C263S) indicates >95% purity (Figure S3A). The 2D ^{1}H-^{15}N HSQC NMR spectrum for a sample of the DUSP5 PD(C263S) protein shows good chemical shift dispersion, indicating the protein is well-folded (Figure S3B). We also obtained 2D ^{1}H-^{15}N HSQC NMR spectra in the presence of 250 μM **RR505** (Figure S3C) and observed cross-peak shifting or exchange broadening, consistent with direct binding of **RR505** to ^{15}N-labeled DUSP5 PD(C263S). To assess the ability of the compounds identified via docking to inhibit DUSP5 PD (WT) activity, a phosphatase assay was developed based on a previously published assay [35,40]. The substrate, pNPP, has been shown to react with a wide variety of phosphatases [41]. The assay was performed in 1-mL quartz cuvettes, at various substrate concentrations, and initial velocities were measured at the wavelength of 405 nm due to the formation of *p*-nitrophenolate. Initial velocity data were fitted to the Michaelis-Menten equation (Figure 3A), yielding a V_{max} of $1.35 \pm 0.02 \times 10^{-3}$ (μmol/min) and a K_m of 7.6 ± 0.4 mM. Since some of the inhibitors to be screened were dissolved in DMSO, the effect of

1%, 2%, and 4% DMSO was investigated by substituting appropriate quantities of DMSO for some of the buffer mixture. Relative rates with and without DMSO were compared and plotted in Figure 3B. DMSO appears to activate the DUSP5 PD reaction, consistent with a previous report for DUSP6 [42]. Thus, to ensure consistent results we performed all assays on compounds dissolved in DMSO while maintaining a fixed concentration of DMSO.

To measure IC_{50} values, a control inhibitor was used, and the assay was performed with the addition of a known broad-spectrum inhibitor of phosphatases, sodium orthovanadate (vanadate) [33]. Initial experiments were performed in 1 mL cuvettes and concentrations of vanadate were varied. Initial velocities from kinetic reads were plotted as a function of the log of vanadate concentration and fitted to equation 3, to obtain the IC_{50} of 88 ± 8 nM (Figure S4).

Development of HTS assay for screening inhibitors and validation

The pNPP phosphatase assay was also performed in a plate format to increase throughput by which inhibitors could be screened. To validate the plate assay, the *Z*' factor [37] was determined using the plate arrangement described above. The absorbance values for the end-point assays and the slopes for the kinetic assays were averaged (summarized in Table 2), and Z' values were calculated using equation 2. Both the end point assay (Z' = 0.73) and the kinetic assay (Z' = 0.74) formats resulted in Z' factors in the acceptable range for an HTS assay.

Compounds identified by protein-based (docking) and ligand-based (ROCS overlays) *in silico* screening methods were tested experimentally using the plate assay described above. Initial velocities were measured for the first 10 minutes of reaction, plotted (Figure 4), and then fitted to equation 3 to obtain IC_{50} values (summarized in Table 1). We tested **SM1842** (**RR505** + **RR506**), **RR505** and **RR506** in the HTS pNPP assay. Triton X-100 was used in this assay (at 0.1%) to disrupt any small molecule aggregates that could be formed. The IC_{50} for **SM1842** is 5.1 mM, while for **RR505** and **RR506** is 26 and 16 mM respectively (Figure 4A; Table 1). Collectively, these data suggest that **SM1842** or its analog mixture identified from the computational docking assay inhibit DUSP5 in the mM IC_{50} range.

Suramin, an FDA approved analog, was identified via lead hopping

To identify molecules with similar shape and electrostatic properties to NTS (IC_{50}: 6.4 mM in pNPP assay) (Figure 4B), we subsequently searched a Drugbank [32,33] (chemical library that contains structures of FDA approved drugs) using ROCS with the **RR505** and NTS structures. Suramin was found as a match to the NTS structure. Indeed, suramin (Table 1) is comprised of two NTS substructures connected via a rigid linker, and is an FDA approved drug that is used to treat African sleeping sickness [43]. Initial velocity inhibition profiles of suramin were obtained by measuring initial velocities at varied concentrations of substrate (pNPP) and inhibitor (suramin) (Figure S5). The inhibition profile fits best to the equation for competitive inhibition (eq. 4).

$$(4)\quad v = \frac{V_{max}[S]}{K_m\left(1+\frac{[I]}{K_i}\right)+[S]}$$

where v is the initial velocity, V_{max} is the maximum velocity, K_m is the Michaelis constant, and $[S]$ is the concentration of pNPP. The data were fitted to the equation for competitive inhibition, to give a K_i of 24.6 ± 5.2 μM (Figure S5). Competitive inhibition suggests that inhibition occurs via specific blockage of the phosphatase active site (suramin competes for pNPP binding in the phosphatase active site). Aggregation is often an issue with small molecules, so we investigated whether suramin aggregates in solution. To test for aggregation, the assay was repeated in the presence of Triton X-100, a detergent that is able to break up small molecule aggregates, while not significantly interfering with the assay [44]. NTS (Figure 5A) and suramin (Figure 5B) inhibition was compared under identical conditions in the absence and presence of Triton X-100. Results show that detergent decreases suramin inhibition, while it has no effect on NTS inhibition. This indicates that at least some of suramin's inhibition is due to aggregation, and that this tendency to aggregate is not an inherent property of just the polysulfonated aromatic group contained within the suramin molecule (NTS). But, while the level of inhibition due to suramin decreases in the

presence of added detergent, it is not eliminated (Figure 5C). This suggests that while suramin aggregates, at least some of its inhibition is not due to aggregation, consistent with the fact that suramin behaves as a competitive inhibitor (nonspecific inhibition would not appear competitive).

We additionally confirmed the potential aggregating effects of suramin and other compounds using nephelometry methods. When an inflection occurs in the Relative Nephelometry Units, measured as a function of compound concentration, this indicates that the particle size in solution is increasing, due to aggregating effects. **RR505**, **RR506**, NTS, and suramin were subjected to nephelometry measurements (Figure S6). Suramin appears to start forming aggregates around 25 μM, while **RR505**, **RR506** and NTS do not appear to form aggregates. This is consistent with the Triton X-100 studies that suggest that suramin inhibition, unlike NTS, is at least partially due to aggregation effects.

Identification of small molecule inhibitors based on distance between sulfates in the DUSP5 PD active site

All compounds identified thus far have had two or more sulfonate groups on a polycyclic aromatic core structure. Recognition of this common theme amongst ligands, and inspection of the active site pocket of the crystal structure of the human DUSP5 PD [16], led us to a hypothesis regarding the required pharmacophore features of DUSP5 PD ligands. Since the DUSP5 PD crystal structure contains two sulfate ions in the active site pocket (Figure 6A) in the regions suggested to be occupied by the di-phosphorylated substrate (pThr-Glu-pTyr, of ERK2), and our ligands generally possessed at least two sulfonates (Table 1), we reasoned that two such negatively charged moieties—appropriately positioned—are a necessary feature of any DUSP5 PD inhibitor. To this end, a ligand-based search strategy based on the two bound sulfate ions was then pursued. These sulfate ions are positioned 7.2 Å from each other (Figure 6A), with one located where the phosphate to be cleaved would reside (this site is called S1), proximal to the Cys263 nucleophile (Figure 6A). The other, termed S2, is located 7.2 Å away, in an arginine-rich pocket. Overlay of compound **RR505** with the S1 and S2 sulfates was less than optimal (Figure 7B), while the NTS overlay was better (Figure 6C). A ROCS search was made of all compounds

in the CSD^3 internal collection for hits that had sulfonates in the proper S1;S2 location, and compound **CSD^3_2320** was identified (Figure 6D). This compound (like NTS) is based on the simpler naphthalene core, and has an IC_{50} of 32.7 $\pm$ 2.3 mM (Figure 7A) in the phosphatase assay with DUSP5 PD(WT) and using pNPP as substrate. But, in the more biologically relevant assay with full length DUSP5 and using pERK2 as substrate, the IC_{50} is in the range of 8-96 μM with a mean of 33 $\pm$ 21 μM (Figure 7B). The 1D ^{1}H NMR spectrum confirms that **CSD^3_2320** is pure (Figure S7) and matches the expected structure; and, nephelometry indicates that it has no propensity to aggregate (Figure S8). NMR HSQC titration experiments (Figure 7C) confirm direct binding to DUSP5 PD(C263S), with several crosspeaks being shifted in the presence of **CSD^3_2320**. When **CSD^3_2320** is positioned in the model of the ERK2-DUSP5 complex (Figure 1) by overlaying the sulfonates on the phosphate groups of the ERK2 activation loop, a close superposition is obtained that positions the phenolic ring of the **CSD^3_2320** naphthalene core where the tyrosine would bind (Figure 7D), with the hydroxyl group in the vicinity of where the incoming water nucleophile would be located, before attack on the substrate's phosphate ester. CSD3_2320 was also screened against a panel of 21 phosphatases and found to not inhibit any of them significantly at up to 30 μM (Figure S9).

Discussion

Docking and HTS assay to identify polysulfated lead molecules

Docking into the phosphatase domain (PD) of the full-length DUSP protein (Figure 1) and ROCS alignment calculations have identified various polysulfonated aromatic compounds, with both carbazole and naphthalene scaffolds (Figure 2; Table 1; Figure 6E). In order to determine affinity of compounds identified using docking studies, an enzyme inhibition (IC_{50}) assay was developed whereby dephosphorylation of pNPP is monitored. pNPP was found to be a substrate for DUSP PD(WT) with a K_m of 7.6 mM (Figure 3), apparently serving as a mimic of the natural substrate, the phospho-tyrosine of ERK2. The DUSP5 PD(WT) IC_{50} assay using pNPP as substrate (Figure 4) was adapted, optimized and

validated as a high throughput screening (HTS) assay, and was found to be suitable for HTS with a Z' value > 0.7 (Table 2). Enzymatic screening of compounds identified by docking identified a number of weak-binding polysulfonated inhibitors that could be used as drug lead scaffolds (Table 1; Figure 6), off of which more potent lead molecules could be developed by rational drug design or by fragment-based drug design techniques, if proximal binding pockets can be identified.

Lead-hopping to an FDA approved drug and beyond

We have also employed a novel approach, to transition from initial lead molecules (**SM1842/RR505** and **RR506**; Figures 2 and 4) to an advanced clinical candidate, by screening for FDA approved compounds that match the shape and electronic properties of a lead molecule, using ROCS. Based on this ROCS overlay, naphthalene trisulfonate (NTS) was found to match the shape and electronic properties of **RR505** (Figure 2B); and, NTS was found to be present in the suramin (Table 1), an FDA approved drug available from Centers for Disease Control for treating African sleeping sickness [43]. Suramin is a competitive inhibitor versus pNPP, binding with a K_i of 25 μM (Figure S5).

While initially promising, suramin does not exhibit properties of a good drug lead molecule, even though it is FDA approved. In particular, while suramin is a reasonably potent competitive inhibitor, it also causes non-specific aggregation. Based on DUSP5 PD(WT) IC_{50} assays performed with and without detergent (Figure 5), along with nephelometry studies (Figure S6), we conclude that while suramin does inhibit by direct binding to the phosphatase domain (Figures 5B and 5C), it also forms aggregates *in vitro* which can lead to additional non-specific protein inhibition effects. This aggregation phenomenon raises more global concerns regarding the current clinical use of suramin, and may in part explain some of the known toxicity associated with suramin [43]. Indeed, literature on suramin [19,44-47] indicates that it can bind to many protein targets, so may lack specificity in its mechanism of inhibition.

DUSP5 inhibition vs. activation: implications for vascular anomalies

We identified the S147P mutation in DUSP5 in patients with vascular anomalies [2], which results in a mutant hypoactive protein [8]. This mutation thus presumably results in increased pERK levels in the "putative causative cell," whose identity is unknown for now. It is presumptive to imply that

this mutation is causative because: (a) most diseases are not the result of a single aberration in a gene product, (b) single gene knockouts in mice and its subsequent phenotype does not necessarily imply causative role in disease, but perhaps the potential functions of the gene product in different tissues, and (c) finally, the etiology of disease, and the context of the mutation in the disease needs to reconciled, which is often not considered. For example, in vascular anomalies such as hemangiomas, which are thought to be inborn errors during embryonic development, there are two phases: the first phase is the increased proliferative phase or the rapid growth phase, and the second phase is the involution or the regression phase. The cellular dynamics, behavior and local milieu in the two phases are likely to be distinct. Whether DUSP5 functions in the early or later phase is not known. Because the proliferative phase is the initial phase, and p-ERK is involved in cell proliferation [5], therefore the natural presumption is that DUSP5 is involved in the first phase. Therefore, our attempts to inhibit DUSP5 could stop the disease in the first phase. However, if inhibiting DUSP5 accelerates the disease in the first phase as the putative tumor suppressor role of DUSP5 would suggest, then, perhaps the involution second phase of hemangiomas could be triggered earlier assuming that the two phases are linked by a common mechanism involving DUSP5. Therefore, the benefit of inhibiting a "putative tumor suppressor," such as DUSP5, and in turn accelerating the disease etiology to a phase where the disease regresses is counterintuitive. It is noteworthy that loss of DUSP5 does increase apoptosis of endothelial cells [2,48], suggesting that DUSP5 as a survival factor for ECs. This perhaps occurs in the regression phase of the hemangioma disease. The debate as to whether to develop activators or inhibitors of DUSP5 is therefore context dependent, and probably both have benefits in specific stages of disease. Irrespective of the strategy, phosphatases as targets for drug discovery present their unique challenges as highlighted in the findings in this manuscript. Although we rationalized on developing DUSP5 inhibitors for vascular anomalies, it is becoming increasingly clear that DUSP5 inhibitors could be viable for other conditions especially those associated with immune system. Recent publications [6, 7] have demonstrated a role for DUSP5 in the immune system. Our unpublished work (Kutty, R, Ramchandran, R. et al.) also supports

these findings. These studies together underscore the importance of DUSP5 in a wide array of phenotypes in different tissue types, with likely more to be discovered in the future.

Charge separation vs. distance hypothesis

While protein-based methods (i.e. docking) have identified a series of weak binding polysulfonated lead molecules (Table 1) for DUSP5, and lead-hopping with ROCS has identified the FDA-approved drug suramin, none of these are viable drug leads without further modification. Thus, more lead molecules and analogs are needed. An interesting feature of all these weak-binding lead molecules is the presence of at least two charged sulfonates, separated by 6-9 Å (Table 1). This led us to hypothesize that this trend is occurring because the active site pocket of DUSP5 PD binds a peptide loop from ERK2 containing two phosphates, so is designed to accommodate two negatively charged functionalities separated by this approximate distance. Indeed, DUSP5 PD was found to crystallize with two sulfate anions bound, at an S-S distance of 7.2 Å (Figure 6). These observations led us to conclude that the key pharmacophore feature for DUSP5 PD binding is two negatively charged groups (such as sulfates or sufonates , tethered by a core scaffold (carbazole and naphthalenes have been identified herein). Negatively charged functional groups are commonly observed on phosphatase inhibitors, but are also associated with poor ability to penetrate cell membranes. Indeed, the polysulfonate compounds identified herein did not show activity in our preliminary assays using human umbilical vein endothelial (HUVEC) cells, which we speculate is due to their inability to penetrate cell membranes. Thus, future studies will be directed to substituting the sulfonates with functional groups that are more likely to penetrate into cells, such as carboxylates, tetrazoles or sulfonamides. Using this pharmacophore feature of two negatively charged groups separated by 7.2 Å in a ligand-based screen, a naphthalene-based disulfonate compound, **CSD^3_2320**, was identified (Figure 7). **CSD^3_2320** has an IC_{50} of only 33 mM if assayed using the phosphatase domain alone (Figure 7A), but 33 μM if assayed using the full-length DUSP5 with ERK2 as substrate (Figure 7B). **CSD^3_2320** is unique, in that it is the only compound tested that showed such a dramatic difference in IC_{50} values when measured in the two assays, indicating that it is especially sensitive to conformational differences that may exist in the binding site pocket in the full

length versus the isolated phosphatase domain. Supporting this argument is the fact that the full length DUSP5 protein also contains an ERK binding domain, tethered via a flexible linker (Figure 1). Also, the native substrate for DUSP5, namely the ERK2 protein, is much larger and capable of a wider range of inter-molecular interactions than the pNPP substrate, which is intended only to mimic the phosphotyrosine of pERK. Thus, while the DUSP5 PD(WT)/pNPP assay is a useful preliminary screen, a subsequent assay using full-length DUSP5 and ERK2 substrate provides the *in vitro* "physiologically relevant" assessment of potency for a lead molecule. Importantly, **CSD^3_2320** shows no tendency to aggregate. Thus, the 7.0-7.5 Å–separated disulfonate is a consistent pharmacophore feature for inhibition of the DUSP5 PD (Table 1), which shows some dependence on the presence of intact DUSP5 protein versus use of just the phosphatase domain. These and other features are part of ongoing studies to further improve the potency of **CSD^3_2320.**

Conclusion

This study illustrates the challenges associated with structure-based drug design applied to dual-specificity phosphatases, which have a preference for highly charged ligands. Screening results presented herein typically yielded polysulfonated aromatic compounds with charged groups separated by ~7.2 Å, and included the FDA-approved drug suramin (Figure 1). While polysulfonated aromatic compounds often aggregate like suramin, careful secondary screens using nephelometry and detergent have allowed for the identification of authentic competitive inhibitors, such as **CSD^3_2320**. The potency of **CSD^3_2320** under the more biologically relevant conditions of the DUSP5 (full-length)/pERK2 assay is 33 μM. **CSD^3_2320**'s sulfonate groups are positioned 7 Å apart, to mimic the two phosphates on the ERK2 tripeptide substrate (pThr-Glu-pTyr). **CSD^3_2320** is a suitable scaffold upon which to build more potent and selective DUSP5 inhibitors; but, in any such inhibitor optimization effort, it will be crucial to perform secondary assays using the full-length DUSP5 protein, and using nephelometry and detergent screens to eliminate compounds that show the nonspecific aggregation effects common to sulfonates.

AVAILABILITY OF SUPPORTING DATA

The data supporting results in this article are included in the article, and in supplementary materials.

LIST OF ABBREVIATIONS USED

CSD^3, Center for Structure-based Drug Design and Development; DMSO, dimethyl sulfoxide; DTT, dithiothreitol; DUSP5, Dual-specificity phosphatase; ERK, extracellular regulated kinase; EBD, ERK binding domain; HSQC, heteronuclear single quantum coherence; isopropyl β-D-1-thiogalactopyranoside, IPTG; LB, Luria-Bertani; MAPK, Mitogen-activated protein kinase; NMR, nuclear magnetic resonance; NTS, naphthalene trisulfonate; PD, phosphatase domain; pERK, phospho-ERK; pNPP, *p*-nitrophenol phosphate; rmsd, root mean square deviation; ROCS, Rapid Overlay of Chemical Structures; WT, wild type; 2D, 2-dimensional.

COMPETING INTERESTS

The authors confirm that they have no competing interests.

AUTHORS' CONTRIBUTIONS

Terrence Neumann: Intellectual contribution to project design and interpretation of results; conducted docking studies, ROCS calculations and NMR titrations; aided in figure development and manuscript preparation.

Elise A. Span: Intellectual contribution to project design and interpretation of results; aided in the design and development of the pNPP assay; implemented the high throughput screen and Z' measurements; conducted PD protein purification.

Kelsey Kalous: Conducted pNPP and nephelometry assays; performed PD protein purification.

Robert Bongard: Intellectual contribution to project design and interpretation of results; conducted screening assays, including IC50 measurements; conducted PD protein expression and purification; Aided in figure and manuscript preparation.

Adam J. Gastonguay: Conducted enzymatic characterization, pNPP assays and western blotting; aided in figure and manuscript preparation.

Michael Lepley: Conducted the Western assay using full-length DUSP5 protein; intellectual contribution to project design and interpretation of results; aided in figure preparation.

Raman G. Kutty: Contributed to the development and optimization of the Western assay using full-length DUSP5 protein; intellectual contribution to project design and interpretation of results.

Jaladhi Nayak: Conducted full-length DUSP5 protein purification and optimization.

Chris Bohl: Conducted DUSP5 PD protein purification and ^{15}N labeling of PD for NMR titrations.

Rachel Lange: Implemented high throughput screen using automation, and performed Z' measurements.

Majher I. Sarker: Performed organic synthesis, purification and characterization of RR compounds.

Marat R. Talipov: Conducted molecular dynamic simulations; intellectual contribution to protein Modeling studies and interpretation of results; aided in manuscript preparation.

Rajendra Rathore: Key role in guiding group members in the organic synthesis and characterization of DUSP5/ERK inhibitors; also in molecular dynamic simulations; aided in manuscript preparation.

Ramani Ramchandran: Key role in guiding group members in the characterization of DUSP5 activity and purification; aided in manuscript preparation.

Daniel S. Sem: Key role in guiding group members in the characterization of DUSP5 inhibition as well as docking and ROCS calculations; aided in all aspects of manuscript preparation.

ACKNOWLEDGEMENTS

RR (Ramchandran) is a recipient of an NIH Vascular Interventions/Innovations and Therapeutic Advances (VITA) award, BAA-NHLBI-CSB-HV-2013-02-JS, that partially supported AGs and MLs salary. DS is partly supported by NIH grants AI101975 and HL112639. JN was partially supported by Lymphatic Malformation Institute. AG, RK and RR (Ramchandran) are partly supported by NIH grants HL090712, HL102745 and HL112639. This research was funded in part by National Science Foundation awards OCI-0923037 and CBET-0521602, for computational resources. Thanks to Andrea Neumann for

the preparation of figures, and members of the DUSP5 team for their valuable insights during the course of this project.

Research Involving Human Subjects

The authors confirm that no research was performed that involved human subjects.

REFERENCES

1. Ferrara N: **Role of vascular endothelial growth factor in physiologic and pathologic angiogenesis: therapeutic implications.** *Semin Oncol* 2002, **29**: 10-14.

2. Pramanik K, Chun CZ, Garnaas MK, Samant GV, Li K, Horswill MA, North PE, Ramchandran R: **Dusp-5 and Snrk-1 coordinately function during vascular development and disease.** *Blood* 2009, **113**: 1184-1191.

3. Caunt CJ, Armstrong SP, Rivers CA, Norman MR, McArdle CA: **Spatiotemporal regulation of ERK2 by dual specificity phosphatases**. *J Biol Chem* 2008, **283**: 26612-26623.

4. Mandl M, Slack DN, Keyse SM: **Specific inactivation and nuclear anchoring of extracellular signal-regulated kinase 2 by the inducible dual-specificity protein phosphatase DUSP5.** *Mol Cell Biol* 2005, **25**: 1830-1845.

5. Caunt CJ, Keyse SM: **Dual-specificity MAP kinase phosphatases (MKPs): shaping the outcome of MAP kinase signalling.** *FEBS J* 2013, **280**: 489-504.

6. Ueda K, Arakawa H, Nakamura Y: **Dual-specificity phosphatase 5 (DUSP5) as a direct transcriptional target of tumor suppressor p53**. *Oncogene* 2003, **22**: 5586-5591.

7. Sumanas S, Jorniak T, Lin S: **Identification of novel vascular endothelial-specific genes by the microarray analysis of the zebrachish *cloche* mutants.** *Blood* 2005, **106**: 534-541.

8. Nayak J, Gastonguay AJ, Talipov MR, Vakeel P, Span EA, Kalous KS, Kutty RG, Jensen DR, Pokkuluri P, Sem DS, Rathore R, Ramchandran R. **Protein expression, characterization and activity comparisons of wild type and mutant DUSP5 proteins**. *BMC Biochem.* 2014, **15**: 27

9. Alonso A, Sasin J, Bottini N, Friedberg I, Friedberg I, Osterman A, Godzik A, Hunter T, Dixon J, Mustelin T: **Protein Tyrosine Phosphatases in the Human Genome.** *Cell* 2004, **117**:699-711.

10. Camps M, Nichols A, Arkinstall S: **Dual specificity phosphatases: a gene family for control of MAP kinase function.** *FASEB J* 2000, **14**: 6-16.

11. Kohno M, Pouyssegur J: **Targeting the ERK signaling pathway in cancer therapy.** *Ann. Med.* 2006, **38**: 200–211.

12. Holmes DA, Yeh JH, Yan D, Xu M, Chan AC: **Dusp5 negatively regulates IL-33-mediated eosinophil survival and function**. *The EMBO Journal* 2014 **24**: 218-235.

13. Moon SJ, Lim MA, Park JS, Byun JK, Kim SM, Park MK, Kim EK, Moon YM, Min JK, Ahn SM *et al*: **Dual-specificity phosphatase 5 attenuates autoimmune arthritis in mice via reciprocal regulation of the Th17/Treg cell balance and inhibition of osteoclastogenesis**. *Arthritis & rheumatology* 2014, **66**:3083-3095.

14. Rushworth LK, Kidger AM, Delavaine L, Stewart G, van Schelven S, Davidson J, Bryant CJ, Caddye E, East P, Caunt CJ *et al*: **Dual-specificity phosphatase 5 regulates nuclear ERK activity and suppresses skin cancer by inhibiting mutant Harvey-Ras (HRasQ61L)-driven SerpinB2 expression.** *Proceedings of the National Academy of Sciences of the United States of America* 2014, **111**:18267-18272.

15. Fan F, Geurts AM, Pabbidi MR, Smith SV, Harder DR, Jacob H, Roman RJ: **Zinc-finger nuclease knockout of dual-specificity protein phosphatase-5 enhances the myogenic response and autoregulation of cerebral blood flow in FHH.1BN rats**. *PloS one* 2014, **9**:e112878.

16. Jeong DG, Cho YH, Yoon TS, Kim JH, Ryu SE, Kim SJ: **Crystal structure of the catalytic domain of human DUSP5, a dual specificity MAP kinase protein phosphatase.** *Proteins* 2007, **66**: 253-258.

17. Zhou B, Wang ZX, Zhao Y, Brautigan DL, Zhang ZY: **The specificity of extracellular signal-regulated kinase 2 dephosphorylation by protein phosphatases.** *J Biol Chem* 2002, **277:**31818-31825.

18. Piserchio A, Warthaka M, Devkota AK, Kaoud TS, Lee S, Abramczyk O, Ren P, Dalby KN, Ghose R: **Solution NMR insights into docking interactions involving inactive ERK2.** *Biochemistry* 2011, **50**: 3660-3672.

19. Farooq A, Chaturvedi G, Mujtaba S, Plotnikova O, Zeng L, Dhalluin C, Ashton R, Zhou M: **Solution structure of ERK2 binding domain of MAPK phosphatase MKP-3: structural insights into MKP-3 activation by ERK2.** *Mol Cell* 2001, **7**: 387-399.

20. Chou & Fasman Secondary Structure Prediction Server [http://www.biogem.org/tool/chou-fasman/].

21. Chou PY, Fasman GD: **Conformational parameters for amino acids in helical, β-sheet, and random coil regions calculated from proteins.** *Biochemistry* 1974, **13**: 211-222.

22. Chou PY, Fasman GD: **Prediction of protein conformation.** *Biochemistry* 1974, **13**: 222-245.

23. Accelrys Software: Pipeline Pilot. v.8.4

24. Molecular Networks: CORINA. v.2.4.

25. Morris GM, Huey R, Lindstrom W, Sanner MF, Belew RK, Goodsell DS, Olson AJ: **AutoDock4 and AutoDockTools4: Automated docking with selective receptor flexibility.** *J Comput Chem* 2009, **30**: 2785-2791

26. Litzkow M, Livney M, Mutka M: **Condor - A Hunter of Idle Workstations.** In *Proceedings of the 8th International Conference of Distributed Computing Systems:* 13-17 Jun 1988; San Jose, CA. 104-111.

27. Frey J, Tannenbaum T, Livny M, Foster I, Tuecke S: **Condor-G: A Computation Management Agent for Multi-Institutional Grids.** *Cluster Comput* 2002, **5**:237-246.

28. Goodsell DS, Morris GM, Olson AJ: **Automated docking of flexible ligands: Applications of autodock.** *J Mol Recognit* 1996, **9**: 1-5.

29. Hawkins PC, Skillman AG, Warren GL, Ellingson BA, Stahl MT: **Conformer generation with OMEGA: algorithm and validation using high quality structures from the Protein Databank and Cambridge Structural Database.** *J Chem Inf Model* 2010, **50**: 572-584.

30. Rush TS III, Grant JA, Mosyak L, Nicholls A: **A Shape-Based 3-D Scaffold Hopping Method and Its Application to a Bacterial Protein-Protein Interaction.** *J Med Chem* 2005, **48**: 1489-1495.

31. Irwin JJ, Shoichet BK: **ZINC - A Free Database of Commercially Available Compounds for Virtual Screening.** *J Chem Inf Model* 2005, **45**: 177-182.

32. Wishart DS, Knox C, Guo AC, Cheng D, Shrivastava S, Tzur D, Gautam B, Hassanali M: **DrugBank: a knowledgebase for drugs, drug actions and drug targets.** *Nucleic Acids Res* 2008, **36**(Suppl 1): D901-D906.

33. Wishart DS, Knox C, Guo AC, Shrivastava S, Hassanali M, Stothard P, Chang Z, Woolsey J: **DrugBank: a comprehensive resource for in silico drug discovery and exploration.** *Nucleic Acids Res* 2006, **34**(Suppl 1): D668-D672.

34. Marley J, Lu M, Bracken C: **A method for efficient isotopic labeling of recombinant proteins.** *J Biomol NMR* 2001, **20**: 71-75.

35. Chernoff J, Li H: **Multiple forms of phosphotyrosyl-and phosphoseryl-protein phosphatase from cardiac muscle: partial purification and characterization of an EDTA-stimulated phosphotyrosyl-protein phosphatase.** *Arch Biochem Biophys* 1983, **226**: 517-530.

36. Swarup G, Cohen S, Garbers DL: **Inhibition of membrane phosphotyrosyl-protein phosphatase activity by vanadate.** *Biochem Biophys Res Commun* 1982, **107**: 1104-1109.

37. Zhang J, Chung TDY, Oldenburg KR: **A Simple Statistical Parameter for Use in Evaluation and Validation of High Throughput Screening Assays.** *J Biomol Screening* 1999, **4**: 67-73.

38. Bevan CD, Lloyd RS: **A high-throughput screening method for the determination of aqueous drug solubility using laser nephelometry in microtiter plates.** *Anal Chem* 2000, **72**: 1781-1787.

39. Teague SJ, Davis AM, Leeson PD, Oprea T: **The Design of Leadlike Combinatorial Libraries.** *Angew Chem Int Ed* 1999, **24**: 3743-3748.

40. Zhang Z, Dixon JE: **Protein tyrosine phosphatases: mechanism of catalysis and substrate specificity.** *Adv Enzymol Relat Areas Mol Biol* 1993, **68**: 1-36.

41. MacKintosh, C. (1993). In D.G. Hardie (Ed.). *Protein Phosphorylation: A Practical Approach*. 221. New York: IRL Press.

42. Mark JK, Aubin RA, Smith S, Hefford MA: **Inhibition of mitogen-activated protein kinase phosphatase 3 activity by interdomain binding.** *J Biol Chem* 2008, **283**: 28574-28583.

43. Zhang Z, Dixon JE: **Protein tyrosine phosphatases: mechanism of catalysis and substrate specificity.** *Adv Enzymol Relat Areas Mol Biol* 1993, **68**: 1-36.

44. Shoichet BK: **Screening in a spirit haunted world.** *Drug Discovery Today* 2006, **11**: 607-615.

45. Morgan HP, McNae IW, Nowicki MW, Zhong W, Michels PAM, Auld DS, Fothergill-Gilmore LA, Walkinshaw MD: **The Trypanocidal Drug Suramin and Other Trypan blue Mimetics Are Inhibitors of Pyruvate Kinases and Bind to the Adenosine Site.** *J Biol Chem* 2011, **286**: 31232-31240.

46. Yamaguchi F, Yamamura S, Shimamoto S, Tokumitsu H, Tokuda M, Kobayashi R: **Suramin is a Novel Activator of PP5 and Biphasically Modulates S100-Activated PP5 Activity.** *App Biochem Biotech* 2014, **172**: 237-247.

47. Waltenberger J. Mayr U, Frank H, Hombach V: **Suramin is a Potent Inhibitor of Vascular Endothelial Growth Factor. A Contribution to the Molecular Basis of its Antiangiogenic Action.** *J Mol Cell Card* 1996, **28**: 1523-1529.

48. Echavarria R, Hussain SN: **Regulation of angiopoietin-1/Tie-2 receptor signaling in endothelial cells by dual-specificity phosphatases 1, 4, and 5**. *Journal of the American Heart Association* 2013, **2**(6):e000571.

FIGURE LEGENDS

Figure 1. *DUSP5 and ERK2 Models*. (A) Model depicting the two domains of DUSP5. This model is comprised of two domains, the ERK binding domain (EBD) and phosphatase domain (PD), and illustrates the relative location of the domains and their connection via a 30 amino acid linker of unknown structure. The homology model of EBD was constructed using the solution structure (21% identity and 35% homology) of human MKP-3 protein (PDB:1HZM) as a template [19]. The phosphatase domain is the previously reported crystal structure (PDB:2G6Z) [16]. The 30 amino acid linker region connecting the two domains was prepared manually, and is of unknown structure. The S147P mutation present in patients with vascular anomalies is shown in green, and arginine-rich basic regions have been identified. **(B) DUSP5 and ERK2 binding model.** DUSP5 (blue) is positioned similarly in respects to panel A with the EBD to the left and PD to the right, wrapping around human ERK2 in yellow. Model was prepared as described in our previous paper [8]. The linker region may have the first 11 amino acids as helical based secondary structure predictions [20-22], although this was only found to be loosely helical after molecular dynamics simulations. The ERK2 (yellow) structure (PDB:3I60) [18] is shown between the DUSP5 domains to illustrate relative shape and size complementarity; and, relative orientation of ERK2 and DUSP5 is based on the molecular dynamics simulation and associated analysis presented in our previous paper [8].

Figure 2. *Docking Results.* (A) Predicted docking pose of **SM1842/RR505** (gold) in DUSP5 PD (blue), using Autodock 4.2. The inset image shows predicted binding position relative to the rest of the protein. The side chains around the bound ligand (mostly arginine guanido groups) are delineated in light turquoise and the catalytic cysteine is displayed in yellow. Three arginine residues are observed around one sulfonate group of **SM1842/RR505**. The calculated binding energy for this pose was -9.69 kcal/mol and had a cluster population of 10. (B) Optimal overlay of **SM1842/RR505** (gold) and naphthalene trisulfonate (NTS, moss green), using OpenEye Scientific Software ROCS v. 3.0 [30]. (C) Lowest energy binding pose for NTS (moss green) in DUSP5 PD (blue), with a calculated binding energy of -8.48

kcal/mol with a cluster population of 7. (D) Second lowest energy binding pose for NTS (seafoam), with a calculated binding energy of -8.21 kcal/mol. (E) Ligplot drawing of **SM1842/RR505** in the DUSP5 PD binding pocket, showing key interactions.

Figure 3. *Michaelis-Menten Kinetics.* (A) Michaelis-Menten plot of DUSP5 PD(WT) initial velocity versus substrate (pNPP) concentration, monitoring production of p-nitrophenolate at 405 nm. Reaction was in 100 mM Tris-HCl (pH 7.5), 100 mM NaCl, 5 mM $MgCl_2$ and 1 mM DTT, and was initiated with enzyme. The line represents a nonlinear least squares fit to equation 1. (B) Enzymatic rate as a function of DMSO concentration (% v/v), and at a fixed level of pNPP (5 mM), with other conditions as in panel (A). Relative enzyme activation represents the rate normalized to that obtained at 0% DMSO.

Figure 4. *IC_{50} Measurements.* (A) DUSP5 PD(WT) initial velocity versus inhibitor concentration, and fitted to equation 3 to obtain IC_{50} values (Table 1). Conditions were as described for Figure 3. (B) Same as panel (A), but comparing suramin and NTS, demonstrating the affinity increase that is obtained due to tethering the NTS fragments.

Figure 5. *Effect of Detergent on Suramin Inhibition*. (A) NTS IC_{50} measurement in the presence and absence of 0.5% Triton, showing no detergent-effect on inhibition of DUSP5 PD(WT) pNPP phosphatase activity. (B) Suramin IC_{50} measurement in the presence and absence of 0.5% Triton X-100 shows a loss of some inhibitory capability in the presence of detergent. (C) Effect of increasing detergent levels (Triton X-100) on rate of DUSP5 PD(WT) in the presence of a fixed concentrations of inhibitor and substrate (at 1 μM suramin and 5 mM pNPP). Detergent removes some, but not all, of suramin's inhibitory effect, showing a plateau level 30% inhibition.

Figure 6. *Pharmacophore-based Identification of DUSP5 PD Inhibitors*. (A) Crystal structure of DUSP5 PD(C263S) [16], showing the two bound sulfate ions in the two anion-binding pockets postulated to be occupied by the two phosphate groups of the ERK2 activation loop (pThr-Glu-pTyr) [16-18]. The anion pocket closest to the catalytic nucleophile (Cys263) is labeled S1, and the distal anion pocket is labeled S2. The S2 anion (sulfate) is stabilized be several arginine residues, while the S1 anion may derive some helix dipole stabilization by virtue of its location at the N-terminal end of a long central

helix. The sulfur to sulfur distance of 7.2 Å defines the DUSP PD pharmacophore as two anionic groups separated by ~7 Å. Overlay of the S1-S2 pharmacophore (two sulfates, shown as purple) on **RR505** (B) indicates a poor match, while overlay on NTS (C) in one of two possible orientations (related by a 180° rotation) is better. (D) A ligand-based search using this pharmacophore identified **CSD^3_2320**, which also matched the S1-S2 sulfate positions well. The overlay in panel (D), as in panel (C), is shown in one of the two possible orientations that optimally align active site sulfate and ligand sulfonate groups. (E) Flow chart summarizing the docking and ROCS alignment procedures used to identify lead molecules. Once SM1842/RR505 was identified from the CSD^3 Library, it was used as a ROCS query and searched against the CSD^3 Library and ZINC Library. NTS was identified from the ROCS search. NTS was used as a ROCS query to search Drugbank, which led to identification of Suramin.

Figure 7. ***CSD^3_2320 binding to DUSP5 PD.*** (A) Dose response curve for **CSD^3_2320** as an inhibitor of the DUSP5 PD(WT) phosphatase activity, using pNPP as substrate. Experimental conditions as in Figures 3 and 4. Chemical structure of **CSD^3_2320** in the insert. (B) Dose response curve for **CSD^3_2320** as an inhibitor of the DUSP5 (full-length protein) phosphatase activity, using pERK2 as a substrate. (C) DUSP5 PD(C263S) ^{1}H-^{15}N HSQC spectrum of DUSP5 PD(C263S) in pH 6.8, 50 mM potassium phosphate, 100 mM potassium chloride buffer. Overlay is of 500 μM ^{15}N-labeled DUSP5 PD alone (black), and in the presence of 500 μM **CSD^3_2320** (red). Potentially important chemical shift perturbations due to binding are indicated using arrows. (D) The model from Figure 1, with **CSD^3_2320** positioned such that its two sulfonate groups are optimally overlaid with the two phosphate groups on the ERK2 pThr-Glu-pTyr peptide. This overlay results in the phenolic ring of the **CSD^3_2320** naphthalene core being superimposed directly on the tyrosine phenol ring of the pThr-Glu-pTyr peptide.

Table 1. Structures, Docking Energies, and IC_{50} Values of DUSP5 PD Inhibitors

Chemical Structure	Docking Energy (kcal/mol)	IC_{50} from Enzyme Assay
**SM1842**	-9.69	5.1 ± 1.9 mM
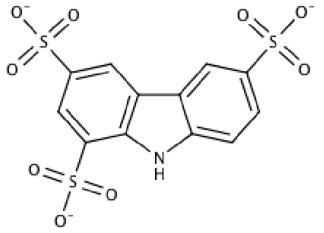 **RR505**	-9.69	26 ± 3 mM
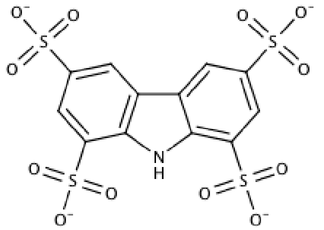 **RR506**	-9.89	16 ± 2 mM
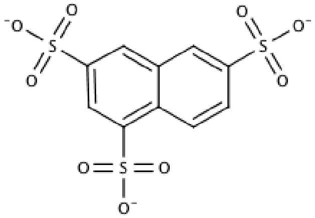 **Naphthalene trisulfonate (NTS)**	-8.48	6.4 ± 0.8 mM*

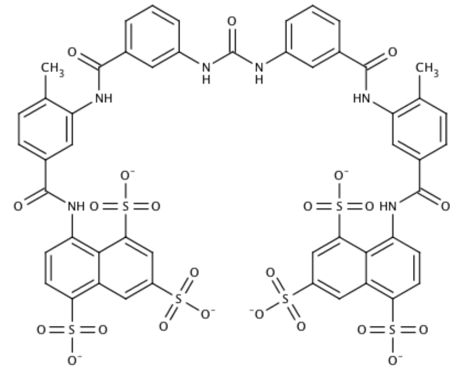 **Suramin**	NA	44 ± 6 μM* (K_i = 25 $\pm$ 5 μM)*

*Obtained in absence of Triton X-100.

Table 2. DUSP5 PD(WT) pNPP enzymatic assay data for Z' calculation (96 well plates)

**A) End-point assay after 90 min**		
	**Positive control**	**Negative control**
**Mean**	0.006	0.269
**Standard deviation**	0.004	0.02
**B) Kinetic (continuous) assay over 5 min**		
	**Positive control**	**Negative control**
**Mean**	-0.035	3.28
**Standard deviation**	0.091	0.193

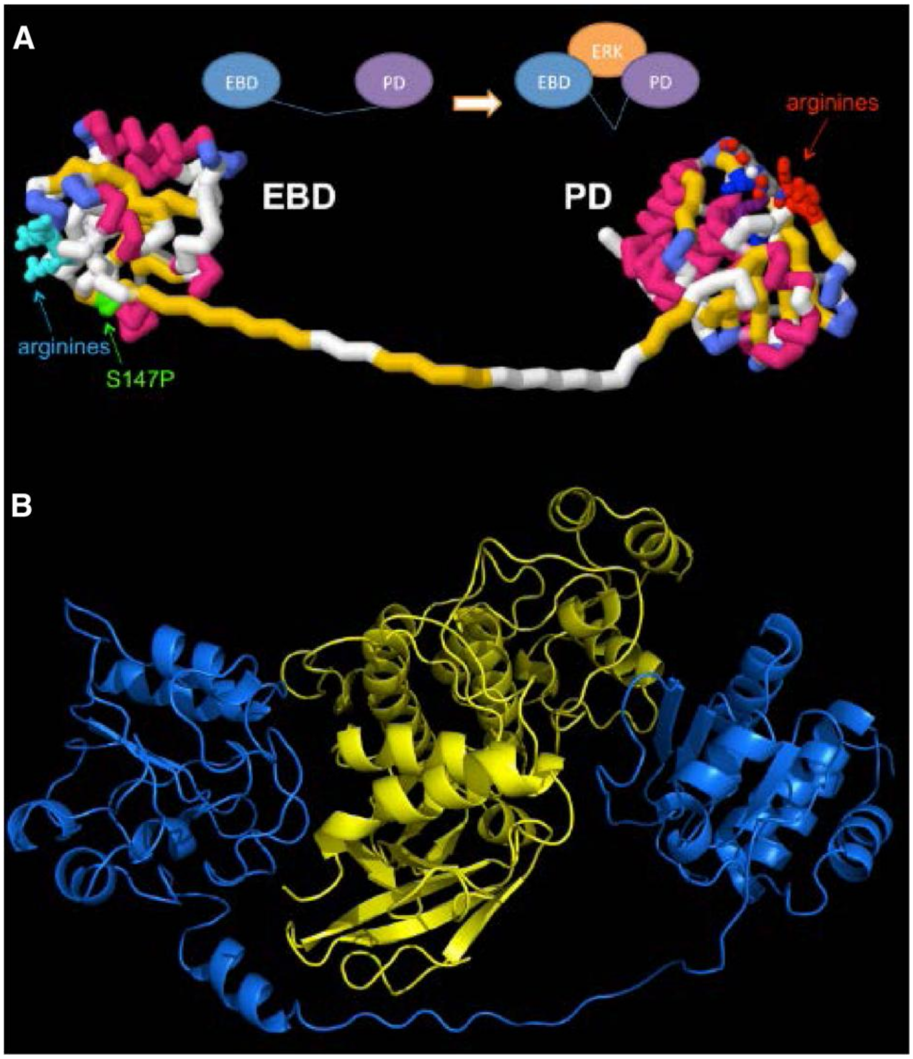
A
EBD
PD
ERK
EBD
PD
arginines
EBD
PD
arginines
S147P
B

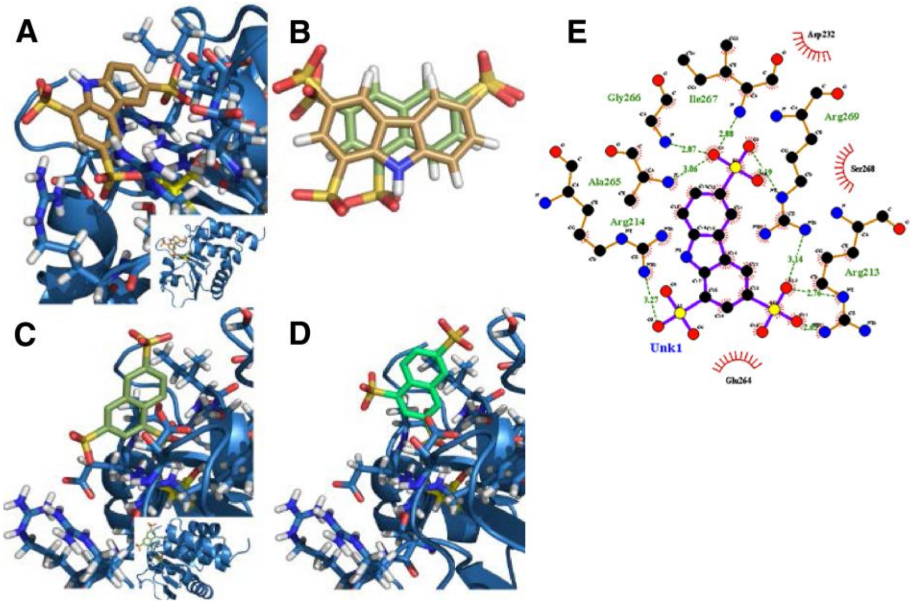
A
B
C
D
E
Asp232
Gly266
Ile267
Arg269
Ser268
Ala265
Arg214
Arg213
Unk1
Glu264

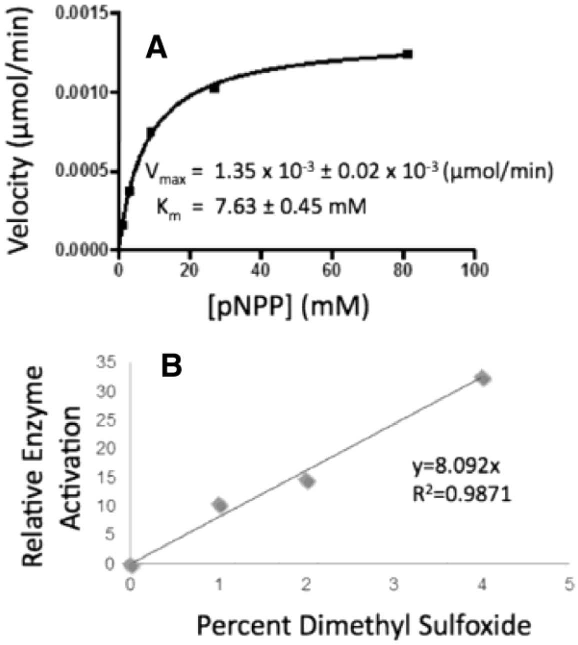
A
Velocity (µmol/min)
0.0015
0.0010
0.0005
0.0000
0
20
40
60
80
100
[pNPP] (mM)
V_max = 1.35 x 10^-3 ± 0.02 x 10^-3 (µmol/min)
K_m = 7.63 ± 0.45 mM
B
Relative Enzyme Activation
35
30
25
20
15
10
5
0
1
2
3
4
5
y=8.092x
R^2=0.9871
Percent Dimethyl Sulfoxide

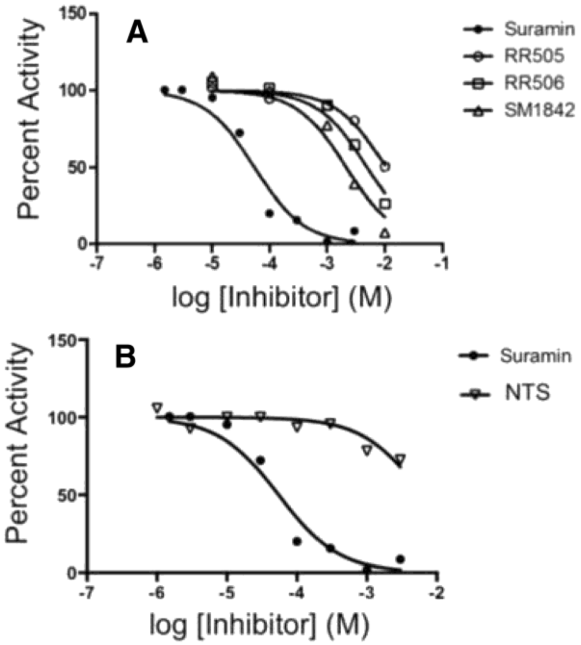
A
Percent Activity
150
100
50
0
-7
-6
-5
-4
-3
-2
-1
log [Inhibitor] (M)
Suramin
RR505
RR506
SM1842
B
Percent Activity
150
100
50
0
-7
-6
-5
-4
-3
-2
log [Inhibitor] (M)
Suramin
NTS

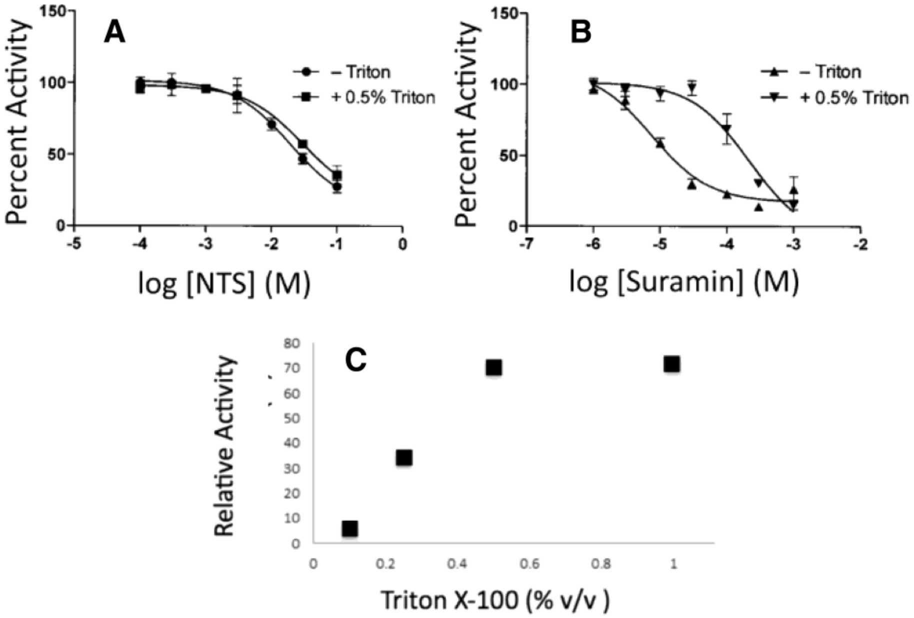
A
Percent Activity
log [NTS] (M)
– Triton
+ 0.5% Triton
B
Percent Activity
log [Suramin] (M)
– Triton
+ 0.5% Triton
C
Relative Activity
Triton X-100 (% v/v)

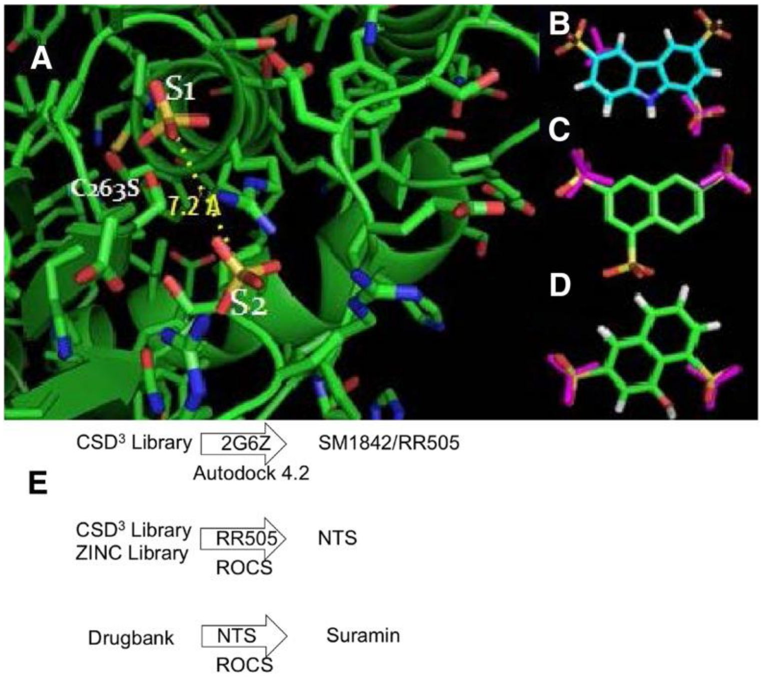
A
S1
C263S
7.2 Å
S2
B
C
D
E
CSD3 Library
2G6Z
Autodock 4.2
SM1842/RR505
CSD3 Library
ZINC Library
RR505
ROCS
NTS
Drugbank
NTS
ROCS
Suramin

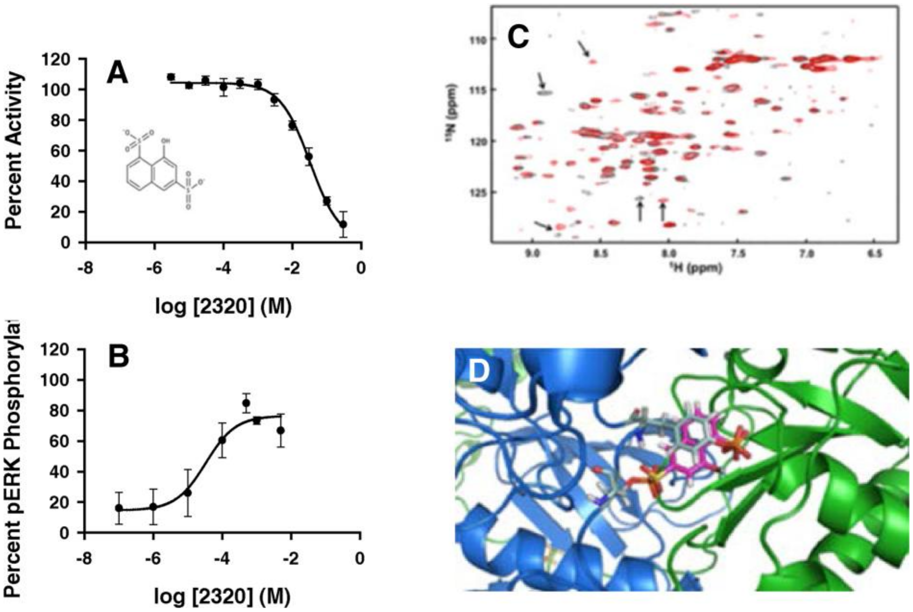
A
Percent Activity
log [2320] (M)
B
Percent pERK Phosphoryla
log [2320] (M)
C
^{15}N (ppm)
^{1}H (ppm)
D
